# Pharmacotherapy of Lower Respiratory Tract Infections in Elderly—Focused on Antibiotics

**DOI:** 10.3389/fphar.2019.01237

**Published:** 2019-10-31

**Authors:** Yang Liu, Yan Zhang, Wanyu Zhao, Xiaolei Liu, Fengjuan Hu, Birong Dong

**Affiliations:** ^1^The Center of Gerontology and Geriatrics, West China Hospital, Sichuan University, Chengdu, China; ^2^Geriatric Health Care and Medical Research Center, Sichuan University, Chengdu, China

**Keywords:** lower respiratory tract infections, elderly, controlled clinical trial, pharmacotherapy, antibiotics, drug resistance

## Abstract

Lower respiratory tract infections (LRTIs) refer to the inflammation of the trachea, bronchi, bronchioles, and lung tissue. Old people have an increased risk of developing LRTIs compared to young adults. The prevalence of LRTIs in the elderly population is not only related to underlying diseases and aging itself, but also to a variety of clinical issues, such as history of hospitalization, previous antibacterial therapy, mechanical ventilation, antibiotic resistance. These factors mentioned above have led to an increase in the prevalence and mortality of LRTIs in the elderly, and new medical strategies targeting LRTIs in this population are urgently needed. After a systematic review of the current randomized controlled trials and related studies, we recommend novel pharmacotherapies that demonstrate advantages for the management of LRTIs in people over the age of 65. We also briefly reviewed current medications for respiratory communicable diseases in the elderly. Various sources of information were used to ensure all relevant studies were included. We searched Pubmed, MEDLINE (OvidSP), EMBASE (OvidSP), and ClinicalTrials.gov. Strengths and limitations of these drugs were evaluated based on whether they have novelty of mechanism, favorable pharmacokinetic/pharmacodynamic profiles, avoidance of interactions and intolerance, simplicity of dosing, and their ability to cope with challenges which was mainly evaluated by the primary and secondary endpoints. The purpose of this review is to recommend the most promising antibiotics for treatment of LRTIs in the elderly (both in hospital and in the outpatient setting) based on the existing results of clinical studies with the novel antibiotics, and to briefly review current medications for respiratory communicable diseases in the elderly, aiming to a better management of LRTIs in clinical practice.

## Introduction

The elderly may suffer from inappropriate medication due to decreased vision, memory loss, impaired cognition, low compliance, and unsupervised care. Hospitalization history, previous antibacterial therapy, physical decline, and mechanical ventilation are risk factors for LRTIs in this population. In the elderly, infections usually manifest as atypical symptoms such as lethargy, loss of appetite and mental disorders, thus inexperienced caregivers tend to ignore the above symptoms leading to the missed diagnosis and inappropriate use of antibiotics, increasing disability and mortality in the elderly. One of the major causes of the growing LRTIs burden is increasing antimicrobial resistance. *Streptococcus pneumonia* (*S. pneumonia*), *Clamydia pneumonia*, *Staphylococcus aureus* and other bacterial pathogens remain the common causes of LRTIs. The resistances of these pathogens to macrolides and fluoroquinolones continue to increase at an alarming rate worldwide (Giske et al., 2008; Woodhead et al., 2011). For example, 48% of US isolates of *S. pneumoniae* tested were macrolide-resistant in 2014 (an increase from the 40% reported in 2008), and high-level macrolide resistance across the US was 33% (Jones et al., 2010). This is also the case in Europe (Ales et al., 2013). Aside from *S. pneumoniae*, atypical LRTIs-causing pathogens, such as *Mycoplasma pneumoniae*, have also produced increased antibiotic resistance (Asche et al., 2008). In the elderly, due to the long-term use of broad-spectrum antibiotics, immunosuppressants and invasive operations increase antibiotic resistance, ultimately leading to excess hospitalizations, treatment failures, and financial burdens. In addition, some physicians are not familiar with the physiological characteristics of the elderly or precautions for common medication, resulting in inappropriate use of antibiotics, such as: 1) Combination therapy with quinolones and warfarin increases the risk of bleeding in elderly patients, leading to QT prolongation; 2) Interaction between macrolides and statins may lead to rhabdomyolysis and acute kidney injury; 3) combination therapies with macrolides, fluoroquinolones, and sulfonylureas may cause severe hypoglycemia in the elderly. 4) Fluoroquinolones, macrolides, sulfonamides, nitrofurans, and β-lactams may cause damage to the central nervous system (CNS); and 5) fungal infections may be associated with the long-term use of antibiotics. In addition, antibiotics may affect the accuracy of diagnostic tests. Due to the high prevalence of LRTIs in the elderly both in hospital and outpatient setting (**Table 1**), the epidemiological differences, atypical clinical manifestations, and age-related variations in pharmacokinetics and pharmacodynamics make LRTIs management for the elderly more challenging, and standardized treatment at early stage of LRTIs is critical to reducing deaths and disability at present.

**Table 1:** Major pathogens and risk factors for pneumonia in community and LTCFs.

Pathogens	Prevalence of CAP in community elderly (%)	Prevalence of pneumonia in LTCFs elderly	Risk factors
*S. pneumoniae* (Mufson and Stanek, 1999; Waterer et al., 2001; Martinez et al., 2003; Baddour et al., 2004)	5–58	4–55	Used lactams, fluoroquinolones, macrolides in the past 3 months; COPD; History of pneumonia in past 12 months; Aspiration.
*H. influenzae* (Lau et al., 2006; Jean et al., 2009; Kuo et al., 2014)	2–29.4	0–22	Severe underlying disease; Used antibiotics in the past 3 months;
*Staphylococcus aureus* (Wunderink et al., 2003; Bernardo et al., 2004; Stevens et al., 2007; Kalil et al., 2013; Bradley, 2014)	0–7	0–33	Hospitalized in the past 3 months; Used antibiotics in the past 3 months; Living in LTCFs; Received intravenous therapy or dialysis for the past 30 days; Confirmed MRSA by etiological diagnosis; Comorbidity; Mental disorders.
*Legionella* (Miller, 1981; Edelstein et al., 1996; Genne et al., 1997; Vergis et al., 2000; Blazquez Garrido et al., 2005; Mykietiuk et al., 2005; Sabria et al., 2005; Haranaga et al., 2007; Varner et al., 2011)	0–17.5	0–6	Smoking; Chronic disease; Immunosuppression; Air conditioning and hot water system use.
*Gram-negative enteric bacilli* (Ortiz-Ruiz et al., 2004; Yakovlev et al., 2006; Peto et al., 2014)	0–12.4	0–14.3	Living in LTCFs; Tube feeding; Comorbidity; Cerebrovascular disease; Dementia; Use of Proton pump inhibitors (PPIs).
*Pseudomonas aeruginosa* (Ding et al., 2016; Francois et al., 2017; Bassetti et al., 2018; Ocheretyaner and Park, 2018; Riquelme et al., 2018)	1–17.1	0–6	Hospitalized in the past three months; Used antibiotics in the past 3 months; Aspiration; Impaired swallowing; Use of PPIs; Structural lung disease or severe bronchiectasis; Confirmed pseudomonas aeruginosa in the past 12 months; Severe illness (requires ventilator or admission of ICU).
*Chlamydia pneumonia* (Arnold et al., 2016; Marchello et al., 2016; Perrone and Quaglia, 2017; Webley and Hahn, 2017)	0–28	0–18	
*Mycoplasma pneumoniae* (Cao et al., 2017; de Groot et al., 2017; Sharma et al., 2017; Waites et al., 2017b)	1–13	1	

According to the 2017 Global Burden of Disease (GBD) Study (James et al., 2018), the burden of LRTIs in people older than 70 years old is still increasing in many regions (Troeger et al., 2017). Altered respiratory structure caused by aging (Song and Chang, 2017), impaired organ function (Poulose and Raju, 2014), changes of drug-susceptibility (Alldred et al., 2010), and chronic low grade inflammation (Boyd and Orihuela, 2011) together lead to the increased susceptibility to LRTIs. Meanwhile, the existing of comorbidities and aging, drug resistance, the prevalence and mortality of LRTIs in the elderly are much higher than other age groups, thus there is a huge demand for the development of novel pharmacotherapy for the elderly, and antibiotics seem to the cornerstone of LRTIs management (Katzan et al., 2003; Ma et al., 2013; Choi et al., 2018).

Based on the existing data of phase 3 clinical trials with the latest antibiotics, the purpose of this article is to recommend the most promising antibiotics for the treatment of LRTIs in the elderly. Meanwhile, we briefly reviewed current medications for respiratory communicable diseases in the elderly, aiming to obtain a better management of LRTIs in clinical practice.

## Methodology

We comprehensively reviewed the research status of medication for LRTIs in the elderly and antibiotics, which are currently in advanced stages of development (phase 3 trial and beyond). After systematically retrieving the following sources including Pubmed, MEDLINE (OvidSP), and EMBASE (OvidSP) from October 2010 to July 2018, we have collected 87 clinical trials and manual screened out 58 trials (thirty-seven Phases 1 and 2 trials, eighteen Phase 3 trials, three Phase 4 trials, respectively), and finally elaborated the advantages and limitations of the application of novel antibiotics in clinical practice based on these trials.

All the random control trials (RCTs) included in our study share the following characteristics: trials included patients over 65 years of age who met at least three symptoms: cough, purulent sputum, dyspnea or pleurisy; if they had at least two abnormal vital signs, had at least one laboratory test result or clinical sign associated with LRTIs, and had radiologically confirmed pneumonia, these trials were classified as the risk classes in Pneumonia Severity Index (PSI), ranging from II to V. All of the trials we included were registered on ClinicalTrials.gov to assess the efficacy and safety of certain antibiotics. Population analysis, end points, and assessments were considered. Analysis populations including the intention-to-treat (ITT) population included all subjects who underwent randomization. The clinically evaluable (CE) population was defined as subjects who survive with resolution or improvement in symptoms and infections that further antibacterial therapy was not required. The microbiologic intention-to-treat (mITT) population was defined as all subjects in the ITT population who had a causative pathogen or pathogens identified at baseline by the culture of blood or respiratory specimens or using a culture-independent method. The clinical per-protocol population was defined as subjects in the ITT population who had a qualifying infection as defined by the trial entry criteria, had received a trial agent, had not received any antibacterial agent that was not as signed within the trial that could confound interpretation of the trial results, and had undergone an assessment of results during the protocol defined window. The microbiologic per-protocol population included the patients in both the clinical per-protocol population and the mITT population. Regarding end points, firstly the primary efficacy end point was evaluated as early clinical response (ECR), which was defined as survival with improvement of one or more levels relative to baseline in two or more symptoms of pneumonia and no worsening of one or more levels in other symptoms of pneumonia, without receipt of rescue antibacterial therapy. Generally, ECR was assessed 24–72 h after the first dose of trial drug in the ITT population. The secondary end point was investigator-assessed clinical response at a post-treatment evaluation 5 to 10 days after the last dose, with clinical response defined as resolution or improvement in signs or symptoms to the extent that further antibacterial therapy was unnecessary. At the same time, we also evaluated the adverse reactions of antibiotics, including mild adverse events, serious adverse events (SAEs) defined as adverse events emerged after treatment initiation, and treatment discontinuation. The mortality in both arms was also analyzed for the safety of certain agents.

## Antibiotics for Community Acquired Pneumonia

### Fluoroquinolone

In recent years, new fluoroquinolone agents (**Table 2**), such as delafloxacin, nemonoxacin and zabofloxacin, have been identified as effective against existing fluoroquinolone-resistant pathogens. These new fluoroquinolone agents target both topoisomerase IV and DNA gyrase with stronger affinities, resulting in inhibition of bacterial DNA replication (Kollef and Betthauser, 2019), reducing mutant selection and toxic side effects, and resulting superior potent activity against the most common community acquired pneumonia (CAP) pathogens (Pfaller et al., 2017c). Delafloxacin is effective against Gram-positive bacteria, including *methicillin sensitive Staphylococcus aureus* (*MSSA*), *methicillin resistant Staphylococcus aureus* (*MRSA*), *Moraxella catarrhalis* (*M. catarrhalis*), and *S. pneumoniae*. While nemonoxacin is effective against Gram-positive bacteria, including multidrug-resistant *S. pneumoniae*, *MRSA*, ertapenem-nonsusceptible *Enterobacteriaceae*, *Legionella*, *Chlamydophila*, and *Mycoplasma*. Antibacterial activity of zabofloxacin against MSSA and MRSA is similar to gemifloxacin, but 2–16 times stronger than that of moxifloxacin and ciprofloxacin (Park et al., 2006).

**Table 2:** Summary of advantages and limitations of the novel antibiotics.

Antibiotics	Mechanism of action	Frequency of interactions	Side effects	Frequency of dosing	Phase of study	FDA/EMA approved	Intravenously or orally	Recommend	MIC_90_ of novel antibiotics compared with existing antibiotics
Nemonoxacin	–Target both topoisomerase IV and DNA gyrase. –New fluoroquinolone.	LOW	TRANSIENT ELEVATION OF AMINOTRANSFERASE.	ONCE DAILY	3	YES	ORAL AND IV	A first-line medication.	–CS-MRSA: The MIC_90_ (큎μg/mL) of nemonoxacin, levoflfloxacin, moxifloxacin are 0.25, 0.25, 0.5, respectively. –CR-*MRSA*: The MIC_90_ (큎μg/mL) of nemonoxacin, levoflfloxacin, moxifloxacin are 0.5, 32, 8, respectively (Barriere, 2014).
Zabofloxacin	–Target both topoisomerase IV and DNA gyrase. –4th generation quinolone (fluoroquinolone).	NOT PROVIDED	–Mild, self-limiting. –gastrointestinal (GI) symptoms and allergic reactions.	Once daily	3	NO	ORAL ONLY	Not recommend.	–*PSSP* & *PISP* & *PRSP*: The MIC_90_ (mg/mL) of zabofloxacin, ciprofloxacin sparfloxacin are 0.03, 2, 0.5, respectively (Barriere, 2014). –*MRSA*: The MIC_90_ (斨μg/mL) of zabofloxacin, moxifloxacin, levofloxacin are 2, 8, and 16, respectively (Mohamed et al., 2019).
Delafloxacin	–Target both topoisomerase IV and DNA gyrase. –4th generation quinolone (fluoroquinolone).	Low	–Favorable AEs profile. –nausea, diarrhea.	Q12h	III (STILL PENDING)	NO	Oral and IV.	Not recommend	–MRSA: The MIC_90_ (mg/L) of delafloxacin, moxifloxacin are 0.004, 0.032 respectively. –MSSA: The MIC_90_ (mg/L) of delafloxacin, moxifloxacin are 0.004, 0.125 respectively (Siala et al., 2016).
Omadacycline	–A unique alkylaminomethyl side chain at the c9 position of the tetracycline	Low	–Mild gastrointestinal symptoms. –CHANGES OF HR AND QT INTERVAL.	Once daily	3	Yes	Oral and IV	–Moderate. –For the elderly without cardiac electrophysiological abnormalities.	–*Chlamydia pneumoniae*: The MIC_90_ (mg/mL) of omadacycline, levofloxacin, moxifloxacin are 0.25, 0.5, 1, respectively (Roblin et al., 1997). –*Mycoplasma pneumoniae* : The MIC_90_ (큎μg/mL) of omadacycline, doxycycline, tetracycline are 0.25, 0.5, 0.5, respectively (Waites et al., 2016).
Solithromycin	–The first fluoroketolide, whichbinds to an additional site on rRNA.	HIGH .	SEVER HEPATIC TOXICITY	Once daily	3	NO	Oral and IV	Not recommend	–*MRSA*: The MIC_90_ (mg/L) of solithromycin, telithromycin, azithromycin, clarithromycin are 2, 4, 2, 8, respectively. –*Legionella pneumoniae*: The MIC_90_ (ကμg/mL) of solithromycin, azithromycin are 0.03, 1, respectively (Waites et al., 2016).
Ceftaroline	A strong affinity for PBPs –Destroy cell wall formation.	Low	Mild and self-limiting	THRICE DAILY	3	Yes	IV ONLY	–Moderate. –For elderly Clearance ≥30 ml/min. –For elderly without QT prolongation.	–*Ceftriaxone-nonsusceptible (NS) s. pneumoniae*: The MIC_90_ (ကμg/mL) of ceftaroline, ceftriaxone are 0.12, ≥2, respectively. –*Amoxicillin-clavulanate-NS s. pneumoniae*; The MIC_90_ (ကμg/mL) of ceftaroline, amoxicillin-clavulanate are 0.12, ≥4, respectively. –Levofloxacin-NS s. pneumoniae; The MIC_90_ (ကμg/mL) of ceftaroline, Amoxicillin-clavulanate are 0.12, ≥1, respectively (Pfaller et al., 2017b).
Ceftobiprole	A strong affinity for the PBPs	Low	Mild and self-limiting	THRICE DAILY	3	NO	IV ONLY	–Not recommend. –Data in some centers were unreliable.	–*Amoxicillin-resistant S. pneumoniae*: The MIC_90_ (ကμg/mL) of ceftriaxone, ceftaroline, ceftobiprole are 0.25, 0.06, 0.06, respectively (Green et al., 2014).
Lefamulin	Inhibit protein synthesis by binding to the bacterial ribosome.	–HIGH, –Interact with azole antifungals and midazolam.	Mild	TWICE DAILY	3	NO	Oral and IV	–Moderate. –For elderly without taking azole antifungals, midazolam.	–*Mycoplasma* pneumoniae, macrolide-susceptible: The MIC_90_ (ကμg/mL) of lefamulin, solithromycin, moxifloxacin, Tetracycline, Doxycycline are 0.02, 0.5, 0.25, 1, 0.25, respectively. –*Mycoplas*ma. pneumoniae, macrolide-resistant: The MIC_90_ (ကμg/mL) of lefamulin, solithromycin, moxifloxacin, tetracycline, doxycycline are 0.02, NA, 0.25, 1, NA, respectively (Waites et al., 2017a).
Pristinamycin	Inhibits protein synthesis by binding to the bacterial ribosome 50s subunit	HIGH	Mild	THRICE DAILY	3	NO	ORAL ONLY	Not recommend	–*MRSA*: The MIC_90_ (ကμg/mL) of pristinamycin, linezolid, vancomycin, teicoplanin are 0.5, 0.5, 2, 2, respectively (Zmira et al., 2005).
Iclaprim	Selectively and potently inhibits dihydrofolate reductase.	Low	Mild	Twice daily	II	NO	IV ONLY	Not recommend (HAP)	Vancomycin-*NS MRSA*: The MIC_90_ (ကμg/mL) of iclaprim, vancomycin are 0.25, > 4, respectively (Huang et al., 2017).
Telavancin	–Interfering transpeptidation, polymerization. –Increases potassium and ATP leakage	–HIGH. –Interact with digoxin, warfarin, benzodiazepines.	Mild	Once daily	4	NO	IV ONLY	Moderate (HAP)	–*MRSA*: The MIC_90_ (ကμg/mL) of telavancin, vancomycin, linezolid, levofloxacin are 0.06, 1, 1, > 4, respectively. –*S. pneumoniae*: The MIC_90_ (ကμg/mL) of telavancin, vancomycin, linezolid, levofloxacin are ≤0.015, 0.5, 1, 1, respectively (Duncan et al., 2017).
Tedizolid	Additional interactions with conserved regions of the ribosomal subunit and the d-ring substituent.	Low	REMAINS TO BE SEEN.	Once daily	3 (UNFINISHED)	NO	IV and oral	Not recommend (HAP)	–*MRSA*: The MIC_90_ (ကμg/mL) of tedizolid, linezolid, vancomycin, Levofloxacin are 0.12, 1, 1, > > 4, respectively (Duncan et al., 2017).
Levofloxacin	Target both topoisomerase iv and dna gyrase	Interact with Warfarin, theophylline, NSAIDs	Phototoxicity, systemic active allergic reactions, hepatotoxicity, severe CNS toxicity	Twice daily	IV	YES	IV and oral	–Moderate. –Good post-marketing response.	See above
Ceftriaxone	A higher affinity for PBPs. –Destroy cell wall formation.	Low	Eosinophilia, leukopenia, thrombocytopenia.	Once daily	IV	YES	IV only	–Moderate. –Good post-marketing response.	See above
Moxifloxacin	Topoisomerase ii, iv inhibitor	Low	Diarrhea, fever, CNS, toxicity	Once daily	IV	YES	IV and oral	–Moderate. –Good post-marketing response.	See above
Amoxicillin	A higher affinity for pbp and can destroy cell wall formation more quickly and effectively	Low	Mild and self-limiting,diarrhea, headache, nausea, anaphylaxis	Thrice daily	IV	YES	IV and oral	–Moderate. –Good post-marketing response.	See above
Linezolid	Interactions with conserved regions of the 23s ribosomal subunit and the d-ring substituent of tedizolid.	Low	Mild and self-limiting,diarrhea, headache, nausea	Thrice daily	IV	YES	IV and oral	–Moderate(HAP) –Good post-marketing response.	See above
Vancomycin	Inhibit the synthesis of bacterial RNA and cell walls, and change the permeability of cell membranes.	Low	–Acute kidney injury–Vestibulocochlear nerve damages	Twice or quartic daily	IV	YES	IV only	–Moderate(HAP) –Good post-marketing response.	See above

Reasons for recommending or not recommending have been marked in capital letters, such as IV, II, YES, NO, SEVER HEPATIC TOXICITY; MIC_90_, the minimal inhibitory concentration required to inhibit the growth of 90% of isolates; CR-MRSA, ciproflfloxacin-resistant and methicillin-resistant Staphylococcus aureus; PSSP, penicillin-susceptible Streptococcus pneumoniae; PISP, penicillin-intermediate S. pneumoniae; PRSP, penicillin-resistant S. pneumoniae; MRSA, methicillin resistant Staphylococcus aureus.

### Nemonoxacin

RCT (NCT01529476) of a phase 3 was conducted in CAP patients receiving nemonoxacin 500 mg or levofloxacin 500 mg orally once daily for 7–10 days. A total of 527 patients (18–70 years old) were randomized to treat with nemonoxacin or levofloxacin. The clinical cure rates at test of cure (TOC) visit were 94.3% for nemonoxacin and 93.5% for levofloxacin in the mITT population. The microbiological success rates were 92.1% for nemonoxacin and 91.7% for levofloxacin in the mITT population. Nemonoxacin was as effective and safe as levofloxacin in the treatment of adult CAP patients in terms of clinical cure rates, microbiological success rates, and safety profile (Yuan et al., 2019). For other phrase 3, non-inferiority trials (NCT02205112, NCT03551210), in which old patients accounted for the majority of the participants, had repeatedly confirmed the safety and efficacy of nemonoxacin in the treatment of CAP (van Rensburg et al., 2010; Chang et al., 2019).

### Values to the Elderly

1) Novel mechanism of action: nemonoxacin targets both topoisomerase IV and DNA gyrase, inhibiting DNA synthesis required to bacterial growth (Li et al., 2015); 2) Frequency of interactions: when the creatinine clearance is <50 mL/min, the dosage of levofloxacin need to be adjusted, while nemonoxacin does not induce or inhibit CYP1A2, 2B6, 2C8, 2C9, C19, and 3A4 isozymes (Cao et al., 2014). No dosage adjustment is required for the elderly with impaired renal or hepatic function. 3) Side effects: unlike other commercially available fluoroquinolone agents (moxifloxacin, levofloxacin), nemonoxacin does not exhibit evidences of phototoxicity, systemic active allergic reactions, significant hepatotoxicity, or severe CNS toxicity (Liang et al., 2013; Zhang et al., 2016). 4) Dosing regimen: In a systemic review and meta-analysis of RCTs demonstrated that compared with 500 mg levofloxacin, nemonoxacin (500 mg or 750 mg) was more safe in cardiac conduction as measured by ECG QTc prolongation (Chang et al., 2019). In addition, a single-dose escalation (nemonoxacin 25–1,250 mg) study shows that there were no clinically significant changes in corrected QT in healthy Chinese volunteers (Luke et al., 2010), but the 750 mg dosage had a significantly higher risk of adverse effects than the 500 mg dosage, so the nemonoxacin 500 mg regimen may be adequate for the treatment of CAP (Roychoudhury et al., 2016). The oral dosage of nemonoxacin is 500 mg once daily while it is 100 mg twice daily for levofloxacin, making nemonoxacin a potential therapy for the elderly with LRTIs.

### Zabofloxacin

Multicenter, non-inferior RCT (NCT01658020) of a phase 3 evaluated the safety and efficacy of oral zabofloxacin (367 mg once daily for 5 days) vs oral moxifloxacin (400 mg once daily for 7 days) in treating acute bacterial exacerbation of chronic obstructive pulmonary disease (COPD); 345 participants with moderate acute bacterial exacerbation COPD were selected. In a subgroup of patients without chronic bronchitis but suffering from LRTIs, antibacterial efficacy of zabofloxacin and moxifloxacin therapies were observed, and the cure rates were 85.9% and 84.2%, respectively. No statistical differences of acute AEs and serious AEs were detected between the two arms (Rhee et al., 2015).

### Values to the Elderly

1) Novel mechanism of action: zabofloxacin can inhibit DNA gyrase and topoisomerase IV, thus inhibiting the bacterial DNA replication (Park et al., 2010). Zabofloxacin shows potent *in vitro* activity against *S. pneumoniae* isolates that caused invasive pneumococcal disease, even levofloxacin-resistant strains (Kwon et al., 2006). 2) Side effects: adverse effects include nausea, hypotension, somnolence, and an increase of blood phosphokinase, which are common and minor and will subside spontaneously. Meanwhile, no QT prolongation was detected (Kocsis et al., 2016). 3) Dosing regimen: dosing regimen is relatively simple, requiring only one dose per day.

### Delafloxacin

RCT (NCT02679573) of a phase 3 on comparison of delafloxacin and moxifloxacin for the treatment of adults with CAP was completed. At present, the results of this trial are still pending. Based on this situation, we do not recommend delafloxacin as a first-line agent for LRTIs in the elderly.

### Tetracycline

#### Omadacycline

Omadacycline (**Table 2**) was a novel once-daily aminomethylcycline antibiotic, and became the second tetracycline antibiotic approved by the FDA in 2018. Omadacycline has antimicrobial activity against Gram-positive, Gram-negative, anaerobic, and atypical pathogens (Dougherty et al., 2019). Omadacycline has a higher coverage against MRSA, penicillin- and multidrug-resistant *S. pneumoniae*, and Vancomycin-resistant enterococci (VRE). Omadacycline also has good activity against *H. influenza*, *M. catarrhalis*, *M. pneumoniae*, *L. pneumophila*, *Enterobacteriaceae*, *Ureaplasma* spp., *Bacillus anthracis*, *Yersinia pestis*, and *Clostridium difficile* (Pfaller et al., 2017a).

A phase 3 trial (NCT02531438) on the efficacy and safety of omacycline for CAP patients had been successfully completed. A total of 772 CAP patients (PSI: II–IV) were randomly enrolled into two groups of the equal size. Patients in the two groups took intravenous omadacycline or moxifloxacin in the first three days, and then transitioned to oral omadacycline or moxifloxacin, respectively. Overall, 41.9% of patients in the ITT populations were older than 65 years old, and 85.4% had PSI risk class of III or IV in this population. Study showed no significant differences between the two arms in terms of ECR, 5–10 days of clinical responses, and incidences of AEs. All the patients who died were older than 65 years old (eight in the omadacycline group and four in the moxifloxacin group). These deaths might be caused by progression of the underlying pneumonia or respiratory compromise, HAP, cardiac or vascular events, and cancer. Neither group had clinically relevant changes from baseline in vital signs, laboratory tests, nor ECG findings. Researchers concluded that deaths in both groups were related to underlying disease rather than these two antibiotics. In summary, the efficacy of omadacycline in the treatment of CAP was not inferior to that of moxifloxacin (Stets et al., 2019).

#### Values to the Elderly

1) Novel mechanism of action: the chemical structure of omadacycline contains a unique alkylaminomethyl side chain at the C9 position of the tetracycline. 2) Frequency of interactions: omadacycline has mild drug interactions and favorable safety profiles. *In vitro*, researchers found that omadacycline does not affect cytochrome P450, and that the most common AEs of omadacycline are gastrointestinal symptoms (2019). No clinically significant differences in omadacycline pharmacokinetics were observed based on age. There is no need for the elderly with impaired or and hepatic function to adjust dose of omadacycline. 3) Dosing regimen: dosing regimen is relatively simplistic as only one dose is needed per day. This regimen greatly reduces the likelihood that an impaired-cognitive patient take repeated medicine or forget to take the medicine.

### Macrolide

#### Solithromycin

Solithromycin (**Table 2**) is a novel 4th generation macrolide. It’s the first fluoroketolide to complete phase 3 clinical trials and show activity against the pathogens associated with LRTIs, including macrolide/penicillin-resistant isolates of *S. pneumoniae*. Solithromycin influence the formation and function of 50S ribosomal subunit, causing the frame-shift mutation during translation (Still et al., 2011). Due to the lack of a cladinose moiety, it does not induce erm(B)-mediated resistance (3Rd et al., 2015). And it is less susceptible to mef(A)-mediated efflux than other macrolides as a result of its increased ribosomal binding and greater intrinsic activity (Darpo et al., 2017a).

One trial (NCT01756339) compared the antibacterial efficacy and safety of oral solithromycin for the treatment of CAP in a 114 central non-inferiority RCTs. During this study, patients were randomly assigned (1:1) to receive either oral solithromycin or moxifloxacin. The results showed that 78.2% participants had an ECR in the solithromycin group compared with 77.9% in the moxifloxacin group, showing equivalent efficacy of solithromycin for the primary endpoint. Subjects over 65 years of age with a history of asthma and COPD had higher success rates for ECR and short term follow-up than those without COPD. In addition, the ECR rate is higher in the 75-year-old group, which may be related to the immunomodulatory effects of solithromycin among all groups (Barrera et al., 2016). In another phase 3 trial (NCT01968733), the efficacy and safety of intravenous-to-oral solithromycin were assessed against intravenous-to-oral moxifloxacin for the treatment of CAP. In this trial, the ECR in the ITT population aged 65–74 years old and older than 75 year old showed non-inferiority of solithromycin for the primary endpoint, respectively. The incidence rate of serious AEs was comparable between groups with no significance (File et al., 2016).

#### Values to the Elderly

Solithromycin has many advantages to be provided for the elderly population. 1) Novel mechanism of action: solithromycin demonstrates increased ribosomal binding in comparison with other macrolides. Meanwhile, as the first fluoroketolide, fluorine contributes to tighter binding and increased activity, and the potential for resistance appears to be low (Darpo et al., 2017b). 2) Frequency of interactions: Due to it is inhibition of the CYP3A isoenzyme pathway it has frequent drug-drug interactions like other macrolides such as erythromycin and clarithromycin. 3) Side effects are mild and relatively low in frequency, however there are concerns of severe hepatic toxicity that require further evaluation (Hook et al., 2015). 4) Dosing regimen: solithromycin also has a simple dosing regimen, with once-daily dosing for the treatment of CAP. For the elderly with poor vision, memory loss, cognitive impairment, and low self-adherence, it’s the preferred choice. Moreover, solithromycin is available in both oral and intravenous (IV) formulation, and is highly potent with effective bacteriostatic properties and eradication rates from a pharmacodynamics (PD) perspective.

### Cephalosporin

Cephalosporins, including ceftobiprole and ceftaroline, is the “new-generation” which is effective against MRSA, MSSA, penicillin-resistant *S. pneumoniae*, *Escherichia coli*, and *Pseudomonas aeruginosa* (Green et al., 2014).

#### Ceftaroline

In 2010, ceftaroline (**Table 2**) was approved by the FDA and European Medicines Agency (EMA) for the treatment of CAP. Its broad-spectrum activity, especially its potent antibacterial activity against Gram-positive bacteria, makes ceftaroline an ideal antibiotic for the treatment of CAP. The efficacy and safety of ceftaroline are established in two milestone studies FOCUS 1 and FOCUS 2. FOCUS 1(NCT00621504) enrolled 613 CAP patients 49.2% of whom were aged ≥65 years old. The experimental group was treated with intravenous ceftaroline 600 mg Q12 h × 5–7 days, and the control group was treated with ceftriaxone and clarithromycin. FOCUS 2 (NCT00509106) recruited 627 CAP patients with the same criteria. Almost half (46.8%) of the patients across both groups were aged ≥65 years old. Both arms took the same intervention as FOCUS 1, and only clarithromycin was not used as adjuvant therapy in FOCUS 2. In both FOCUS 1 and 2, ceftaroline and ceftriaxone were well tolerated, with similar rates of AEs, serious AEs, deaths and discontinuations (File et al., 2011; Low et al., 2011). Another published RCT (NCT01371838) included 771 Asian CAP (PORT risk class III–IV) patients meeting the same criteria as FOCUS. The experimental group used exactly the same intervention as in FOCUS, and the control group used double dosage of ceftriaxone. The results show that ceftaroline is superior to ceftriaxone in clinically evaluable (CE) and mITT population. There was no significant difference in safety between the two agents (Zhong et al., 2015). A Phase 4 multicenter study (NCT01666743) was proposed to specifically evaluate the safety and efficacy of ceftaroline in the treatment of CAP in patients 65 years of age, but the study was withdrawn for unknown reasons. Other studies on the safety and efficacy of ceftaroline for CAP are being recruited (NCT02735707) or have not yielded results (NCT03025841).

#### Values to Elderly

(1) Novel mechanism of action: compared with other penicillin or cephalosporin β-lactam antibiotics, ceftaroline has a higher affinity for penicillin-binding proteins (PBPs) and can destroy cell wall formation more quickly and effectively (Justo et al., 2015). Its broad-spectrum activity, especially its potent antibacterial activity against resistant Gram-positive bacteria, makes it an ideal drug for the treatment of CAP. (2) Frequency of interactions and side effects: side effects of solithromycin are mild, and the frequency was relatively low. For elderly patients with moderate impaired renal function, ceftaroline does not require dose adjustment. (3) Dosing regimen: regimen is simple, and intravenous infusion twice a day is sufficient.

#### Ceftobiprole

Ceftobiprole (**Table 2**) has good activity against Gram-positive pathogens. It has species-dependent activity against Gram-negative pathogens (Curcio, 2014).

Two large scale *in vitro* studies (Farrell et al., 2014; Hodille et al., 2017) of ceftobiprole showed that ceftobiprole had strong activity against *MSSA* (100%, 100% susceptible, respectively), MRSA (98.3%, 99.3% susceptible, respectively), *S. pneumoniae* (99.3%, 99.7% susceptible, respectively), and the majority of *Enterobacteriaceae* (87.3%, 82.5% susceptible, respectively). The potency of ceftobiprole against *P. aeruginosa* (64.6%, 72.7% susceptible, respectively) was similar to that of ceftazidime (Kresken et al., 2011). For elderly people in long-term care facilities (LTCFs), agents are necessary for the coverage of rare pathogens, while ceftobiprole has good antibacterial activity against common pathogens of LTCFs, such as *Enterobacteriaceae* and *P. aeruginosa*. Nowadays, ceftobiprole is approved in several European countries for the treatment of CAP and HAP (excluding VAP) (Scheeren, 2015).

The safety and efficacy of ceftobiprole have been demonstrated in two phase 3 trials on patients with CAP and HAP (excluding VAP). The first study (NCT00326287) demonstrated that intravenous ceftobiprole had equivalent efficacy to ceftriaxone with or without linezolid. Details: clinical cure rates for CAP patients were 86.6% vs 87.4% (clinical evaluate population, 95%CI, −6.9, 5.3), and 76.4% vs 79.3% (ITT population, 95% CI, −9.3, 3.6). Pneumonia-specific mortality within the first 30 days was very low in both groups. In addition, common and serious AEs in the ceftobiprole arm were mild and comparable to those in the ceftriaxone arm (Nicholson et al., 2012). The second RCT (NCT00210964) demonstrated ceftobiprole was non-inferior to ceftazidime with or without linezolid. It is worth noting that cure rates for VAP patients were 23.1% vs 36.8% and 37.7% vs 55.9%, suggesting that ceftobiprole was unsuitable for the treatment of VAP (Awad et al., 2014). A retrospective study of the above RCTs evaluated the early clinical improvement in subgroups of high-risk patients. In some subgroups of high-risk patients with CAP (such as patients over 75 years old, or or CAP patients with COPD, or HAP patients with more than 10 baseline comorbidities), particular and significant results were observed that seemed to favor the ceftobiprole over comparators (Pooley et al., 2014).

#### Values to Elderly

(1) Novel mechanism of action: ceftobiprole with a strong affinity for the PBPs, is responsible for the antibacterial activity of staphylococci and pneumococci (Falco et al., 2018). For pneumonia patients with comorbidities, ceftobiprole with the strong bactericidal effect can quickly improve clinical symptoms and ensure a better prognosis. (2) Frequency of interactions: ceftobiprole elimination is not expected to be significantly affected, as this is a minor elimination route, but dose adjustment is necessary for subjects with the renal impairment (Pfaller et al., 2019). (3) Side effects: for comorbid patients older than 75 years old, the incidence of adverse events caused by ceftobiprole is similar to that of non-high-risk patients, suggesting that ceftobiprole is safe and effective for high-risk groups. In addition, ceftobiprole is less likely to cause an antibiotic-related intestinal flora disorder (Horn et al., 2017).

### Pleuromutilin

#### Lefamulin

Lefamulin (**Table 2**) is a potent semi-synthetic antibacterial agent belonging to a novel class known as the pleuromutilins. Lefamulin’s *in vitro* antibacterial profile includes the most important bacterial pathogens causing LRTIs. The antibacterial spectrum comprises *S. pneumoniae*, *H. influenzae*, *M. catarrhalis*, the atypical respiratory pathogens, *MRSA*, β-haemolytic *streptococci*, and *Enterococcus faecium* (Waites et al., 2017a; Veve and Wagner, 2018). Moreover, as demonstrated in cross-resistance studies, lefamulin remains active against clinical isolates resistant to the following antibiotics: macrolides, lincosamides, streptogramin B, oxazolidinones, tetracyclines, β-lactams, quinolones, trimethoprim-sulfametoxazole, mupirocin, and vancomycin (Mendes et al., 2019; Paukner et al., 2019).

The phase 3 clinical trial, LEAP1 (NCT02559310), for evaluating the safety and efficacy of lefamulin for the treatment of CAP has been completed. Participants with CAP were randomized 1:1 to receive lefamulin at 150 mg IV every 12 h or moxifloxacin at 400 mg once daily. After six doses, patients could be switched to an oral administration if pre-specified improvement criteria were met. If *MRSA* was suspected, linezolid was added to moxifloxacin. In LEAP1, patients aged over 65 years old accounted for 47.8% and 39.3% of the lefamulin and moxifloxacin groups, respectively. At this age, lefamulin was non-inferior to moxifloxacin for ECR, or investigators assessed clinical response (IACR). Lefamulin has a low incidence of drug resistance and minimal cross-resistance with other types of antibiotics, making it a new monotherapy for elderly CAP (File et al., 2019). The oral dosage form of lefamulin is under the investigation in LEAP 2 (NCT02813694), and the primary endpoint is similar to LEAP 1. A major difference in study design includes the use of only oral drugs without the addition of linezolid in the moxifloxacin group. The LEAP 2 results are expected to be available in the second half of 2019.

#### Values to Elderly

In LEAP 1, patients ≥65 years of age accounted for 47.8% and 39.3% of the lefamulin and moxifloxacin groups, respectively. At this age, lefamulin was non-inferior to moxifloxacin for ECR or IACR. (1) Novel mechanism of action: inhibit protein synthesis by binding to the bacterial ribosome 50S subunit (Veve and Wagner, 2018), which ensures that lefamulin has a low incidence of drug resistance and minimal cross-resistance with other types of antibiotics, making it a new monotherapy for elderly CAP. (2) Frequency of interactions: lefamulin has little inhibitory effect on CYP3A, however, it’s worth noting that its high protein binding capacity could lead to drugs interaction (Waites et al., 2017a). (3) Side effects: lefamulin only has mild side effects and is highly effective against common CAP pathogens (Mendes et al., 2019).

### Streptogramins

#### Pristinamycin

Pristinamycin (**Table 2**) is a streptococcal-type antibiotic produced by *Streptomyces faecalis*. It inhibits protein synthesis by binding to the bacterial ribosome 50S subunit (Nespoulous et al., 2018). Pristinamycin has strong antibacterial activity against MRSA, MSSA, *H. influenzae*, and *S. pneumonia* (Cooper et al., 2014). In addition, pristinamycin has a synergistic antibacterial effect with vancomycin (Reid et al., 2010).

A phase 4 study (NCT02332577) intended to evaluate the safety and efficacy of pristinamycin in the treatment of mild CAP is expected to be completed in May 2021.

#### Values to Elderly

It is noteworthy that the above trials excluded patients with moderate and severe CAP, which may limit its generalizability. In addition, pristinamycin has only oral formulation, so it’s unlikely that it will ever have a role in treating old patients with severe CAP. We do not recommend pristinamycin as a promising treatment for CAP.

## Antibiotics for Hospital Acquired Pneumonia or Ltcfs Acquired Pneumonia

### Dihydrofolate-Reductase Inhibitor

#### Iclaprim

Iclaprim (**Table 2**) is a broad-spectrum diaminopyrimidine antibiotic that inhibits the dihydrofolate reductase and does not cross react with human enzyme (Laue et al., 2007). Iclaprim is being developed to treat serious respiratory infections, such as hospital acquired pneumonia (HAP), attributed to multidrug-resistant Gram-positive pathogens and cystic fibrosis caused by *S. aureus* (Huang et al., 2018; Huang et al., 2019). Until now, only one phase 2 clinical trial (NCT00543608) has focused on exploring iclaprim’s efficacy on HAP caused by Gram-positive bacteria, but the trial has not been completed. Therefore, iclaprim is not recommended as a routine treatment for elderly HAP.

### Lipoglycopeptides

#### Telavancin

Telavancin (**Table 2**) is a novel semi-synthetic lipoglycopeptides that is active against multidrug resistant (MDR) staphylococci, enterococci, and streptococci. Telavancin was approved by the FDA in 2013 for the HAP and ventilator-associated bacterial pneumonia (VABP). Telavancin has high antibacterial efficacy against *S. aureus* (MIC_90_ = 0.5 mg/L), *S. epidermidis* (MIC_90_ = 0.5 mg/L) (both *MSSA* and *MRSA*), *VISA* (MIC_90_ = 0.5 mg/L), *Streptococcus* (MIC_90_ = 0.03mg/L), and *VanB protein enterococcus*(MIC_90_ = 2 mg/L), but has a poor effect on *VRSA* (MIC_90_ = 8 mg/L) and VanA protein, enterococcus (MIC_90_ = 8 mg/L) (Hassoun et al., 2017). Two RCTs named “ATTAIN” enrolled in more than 700 HAP patients who were randomized to receive telavancin (10 mg/kg, QD) or vancomycin (1 g, Q12H). The results of the study indicate that telavancin was no worse than vancomycin in terms of the clinical cure rate of TOC visits in both ATTAIN studies. The subgroup analysis also showed that telavancin had a better effect on simple *S. aureus* infection, while vancomycin had a better effect on mixed infection of Gram-positive and Gram-negative bacteria. For MRSA, telavancin and vancomycin have similar effects and similar rates of AEs, but telavancin causes a higher proportion of people with elevated serum creatinine levels than vancomycin (10% vs 8%) (Barriere, 2014). In summary, ECG monitoring is necessary for elderly patients with a history of QT prolongation. At the same time, patients using telavancin should be monitored for coagulation parameters before and after dosing (Al Jalali and Zeitlinger, 2018).

#### Values to Elderly

1) Novel mechanism of action: telavancin has a dual antibacterial mechanism of action, which is to inhibit bacterial cell wall synthesis by interfering with cross-linking (transpeptidation) and polymerization (Rubinstein et al., 2011). Meanwhile, telavancin can cause cell death by increasing membrane permeability, resulting in the leakage of intracellular potassium and ATP. 2) Frequency of interactions: telavancin has mild inhibitory effect on CYP3A (Das et al., 2017), thus it can also be used in elderly with hepatic dysfunction. However, it’s worth noting that its high protein binding capacity could lead to drug to drug interaction (Al Jalali and Zeitlinger, 2018). 3) Side effects: telavancin only has mild side effects and high potency for common CAP pathogens. 4) Dosing regimen: the single-dosage or two-dosage regimen can greatly improve the compliance of old patients. In addition, compared with vancomycin, telavancin has the advantages of potent antibacterial activity against *MRSA*, *VISA* and even *VRSA* as well as long half-life. It has good antibacterial activity. It can fill in gaps when vancomycin is resistant (Barriere, 2014). Based on all the above details, we moderately recommend telavancin as a promising antibiotic for LRTIs in elderly.

### Oxazolidinone

#### Tedizolid

Tedizolid (**Table 2**) was approved by the FDA for the treatment of acute bacterial skin and skin structure infections (ABSSSIs) in 2016. Tedizolid is one of very few prospective agents with a spectrum of activity against MRSA and VRE, which are common pathogens in nosocomial pneumonia (Flanagan et al., 2013). Tedizolid shares many structural features with linezolid and has increased antimicrobial potency than linezolid. Many studies have confirmed that the antibacterial potential of tedizolid for linezolid-susceptible and linezolid-resistant Gram-positive pathogens is much higher than that of linezolid (Brown and Traczewski, 2010). To date, no phase 3 trials assessing efficacy of tedizolid for the treatment of HAP have been completed. Until now, no documented short-term animal and clinical studies have reported neuropathies or thrombocytopenia associated with tedizolid, but the safety of tedizolid for long-term administration remains to be seen.

A randomized phase 3 study (NCT02019420) of the safety and efficacy of tedizolid in comparison with linezolid in patients with HAP and VAP is currently ongoing. The primary endpoint is to determine the non-inferiority (NI) in all-cause mortality (ACM) within 28 days after the randomization of intravenous tedizolid phosphate compared with intravenous linezolid in the ITT Analysis Set in ventilated participants with Gram-positive nosocomial pneumonia. The result is expected to be completed by February 2018, but the researchers have not announced the results of the trial.

#### Values to Elderly

1) Novel mechanism of action: additional interaction with conserved regions of the ribosomal subunit and the D-ring substituent of tedizolid contributes to its strong antibacterial potential. The level of tedizolid penetration into epithelial lining fluid (ELF) and alveolar macrophages (AM) is much higher than free-drug exposures in plasma (Housman et al., 2012). 2) Side effects: in the presence of linezolid resistance or hematologic side effects (Lodise et al., 2016), tedizolid is a better choice. 3) Frequency of interactions: for the elderly with any degree of hepatic and renal dysfunction, no dose adjustment was warranted in elderly to achieve therapeutic goals. 4) Dosing regimen: in addition, its better bioavailability, food-independent efficacy, and simple dosing regimens that support once daily administration, making tedizolid popular with clinicians.

## Special Consideration

We briefly review the current status of pharmacotherapies for special types of LRTIs in elderly. We searched the following sources including Pubmed, MEDLINE (OvidSP), EMBASE (OvidSP), from July 2015 to July 2018. We finally concluded that the risk of LRTIs is much higher in immunocompromised old adults with diabetes than healthy elderly. Pharmacotherapies for old patients with special types of LRTIs (fungal pneumonia, respiratory HCoVs, influenza) are basically the same as for all age groups, but at the same time, considering the health status (frailty, long-term lying in bed, recurring infection and excess hospitalization, cognitive impairment), comorbidities, medication and vaccination history are also important for developing individualized medication regimens.

### Diabetes Mellitus

A retrospective study of patients with diabetes reveals a high correlation between prevalence of infection and fasting blood glucose (FBG) in the elderly (Rayfield et al., 1982). In addition, among patients admitted to hospital for LRTIs, the admission rate of patients with diabetes (Winterbauer et al., 1969; Kornum et al., 2007; Peleg et al., 2007; Casqueiro et al., 2012), risk of complications (Peleg et al., 2007) and mortality (Fine et al., 1996; Kornum et al., 2007) were significantly higher than patients without diabetes. Double hit from an aging immune system, host defense may be impaired in diabetes together increase the risk of bacterial, mycobacterial, fungal and viral infections. Furthermore, respiratory dysfunction and microangiopathy together lead to a higher morbidity and mortality in diabetes elderly (Kornum et al., 2007).

Antimicrobial pharmacotherapy for elderly with diabetes is the same as for all age groups (Mandell et al., 2003). Data suggest that elderly patients receiving aminoglycosides have worse outcomes (Gleason et al., 1999), and medication regimen should be individualized, taking into account the patient’s recent antibiotic medication history, comorbidities, suspected aspiration, suspected pseudomonas infection and β-lactam allergy. For pneumonia patients with diabetes, patients who have not recently used antibiotics can take advanced macrolides or a respiratory fluoroquinolone. By contrast, Patients who have used antibiotics recently can choose fluoroquinolone and advanced macrolides. The chronic use of inhaled glucocorticoids in elderly is associated with the increased risk of diabetes, physicians should be aware of this in order to select those patients in whom the benefits will outweigh the risks (Battaglia et al., 2015). At the same time, it is also important for the management of blood glucose level in infected patients. Meanwhile, diabetic patients usually have varying degrees of impaired renal function, antibiotics with nephrotoxicity should be avoided.

### Fungal Pneumonia

Pulmonary fungal infections can occur in old patients with normal or impaired immune function. The morbidity and mortality of fungal pneumonia among the elderly have increased significantly in recent years. The reason is the increase in patients with malignant tumors, as well as organ transplants or autoimmune diseases, resulting in an increase in patients with immunocompromise, leading to an increase in the incidence of fungal pneumonia (Limper et al., 2011; Chen et al., 2018).

Candida pneumonia is rare; in fact, the isolation of Candida from respiratory secretions is of no clinical significance in most cases (Chen et al., 2018). For immunecompetent pulmonary cryptococcosis hosts, fluconazole or itraconazole are recommended, while immunocompromised hosts are recommended to be treated with amphotericin B in combination with flucytosine, and then followed by fluconazole or itraconazole (Li et al., 2017). In patients with normal immune function, patients with pulmonary aspergillosis are recommended to inhale glucocorticoids and bronchodilators and leukotriene receptor antagonists (Denning et al., 2016), while immunocompromised patients with invasive pulmonary aspergillosis are advised to take oral fluconazole or itraconazole or intravenous amphotericin B (Blanchard et al., 2018). For elderly patients with immunodeficiency, intravenous caspofungin or micafungin is recommended, then followed by oral fluconazole or itraconazole (Bao et al., 2017), meanwhile oral administration of posaconazole at the beginning of treatment is another choice (Clark et al., 2015).

### Respiratory HCoVs

Severe acute respiratory syndrome (SARS) and Middle East respiratory syndrome (MERS) are single-stranded, enveloped, positive-sense RNA viruses. Age and underlying disease are pivotal independent predictors of miscellaneous adverse outcomes in SARS (Chan et al., 2003). SARS cases were mainly seen in young healthy individuals, but patients over 60 years old have the highest mortality, whereas half of the cases of MERS-CoV infections occurred in individuals over the age of 50 (Chan et al., 2003; Assiri et al., 2013). There is no difference in treatment options between the elderly and other age groups. Currently, the most commonly prescribed antiviral regimens are ribavirin, IFNs and lopinavir/ritonavir (Morgenstern et al., 2005; Al-Tawfiq et al., 2014; Omrani et al., 2014).

Ribavirin is a nucleoside analogue with broad-spectrum antiviral activity by inhibiting viral RNA synthesis and mRNA capping (von Grotthuss et al., 2003). The efficacy of ribavirin alone or in combination with IFN-β for the treatment of SARS is inconsistent and controversial (Chu et al., 2004; Leong et al., 2004), and Canada announced a ban on ribavirin for the treatment of SARS due to the reported side effects and inadequate efficacy (Chiou et al., 2005). Lopinavir and ritonavir are protease inhibitors that may inhibit the 3C-like protease of MERS, they improve clinical outcome compared with ribavirin alone in SARS patients (Chan et al., 2006; Stockman et al., 2006). There are still no commercial vaccines available against MERS-CoV (Hart et al., 2014). Multiple vaccine candidates targeting the S protein, which is responsible for viral entry, have been developed, including subunit vaccines (Wang et al., 2015; Tai et al., 2017) recombinant vector vaccines (Kim et al., 2014; Gilbert and Warimwe, 2017), and DNA vaccines (Al-Amri et al., 2017; Chi et al., 2017). Other agents, such as mycophenolic acid (MPA), which prevent replication of viral RNA, have showed strong inhibition activity against MERS-CoV *in vitro* studies (Hart et al., 2014). In addition, passive immunotherapy using human plasma was also applied in the treatment of SARS and MERS (Arabi et al., 2015; Mair-Jenkins et al., 2015). Generally, corticosteroids are widely used along with ribavirin during SARS outbreaks (Lee et al., 2004). A variety of other agents, including antiviral peptides, monoclonal antibodies, cellular or viral protease inhibitor may be promising agents for vitro and/or animal models (Ohnuma et al., 2013; Tao et al., 2014; Agrawal et al., 2016; Zumla et al., 2016). But the efficacy in patients with SARS and MERS needs further clinical validation. In *in vitro* experiments, IFN products were effective in inhibiting both SARS-CoV and MRES-CoV152 (Morgenstern et al., 2005; Chan et al., 2013). Meanwhile, although specific antivirals for MERS-CoV and SARS-CoV are developing, medication with repurposing potential, such as loperamide (de Wilde et al., 2014), chloroquine (Keyaerts et al., 2004), cyclophilins (Stamnes et al., 1992), kinase inhibitors (Dyall et al., 2014), may present as additional therapeutics for future coronaviruses.

### Influenza

Influenza-related deaths gradually increase with increasing age (Yu et al., 2013). From 1979 to 2001, adults ≥65 years old accounted for approximately 60% of influenza-related hospitalizations (Casey et al., 2010; Nicoll, 2010). Data from central and south America (Cheng et al., 2015), European Centre for Disease Prevention and Control (ECDC) - Surveillance and Communication Unit (2011), Africa (Cohen et al., 2018), and southeast Asia (Wong et al., 2006; Park et al., 2016; Ang et al., 2017) are consistent, reporting higher morbidity and mortality in old adults.

Due to doubts about the potency of influenza vaccines, the vaccination rate of influenza vaccine among the elderly is very low (Schmid et al., 2017). In addition, insufficient supply of vaccine and vaccine hesitancy also contribute to inadequate vaccination for the elderly.

Some standard-dose (SD) influenza vaccine studies among elderly have estimated benefits in preventing hospitalization and mortality due to pneumonia (Nichol et al., 2003; Nichol et al., 2007; Jansen et al., 2008). Meanwhile, the high-dose (HD) trivalent influenza vaccine (TIV) was 22% more effective than SD influenza vaccine at preventing probable influenza infections, and 22% more effective than SD influenza vaccine in preventing influenza hospital admission (Izurieta et al., 2015). Another retrospective cohort of U.S. veterans found that, in the 85-year-old group, there was a significant reduction in hospitalizations influenza and pneumonia associated with the HD TIV injection (Richardson et al., 2015). According to observational studies and RCTs, HD TIV (Wong et al., 2006; Park et al., 2016), MF-59-adjuvanted influenza vaccine appeared to have efficacy for clinical influenza (i.e., ILI) and serologically confirmed influenza in adults older than 60 years old (Govaert et al., 1994; Engler et al., 2008; Taylor et al., 2012; Van Buynder et al., 2013; Darvishian et al., 2017; Domnich et al., 2017; Shay et al., 2017). For the diagnosed Influenza, neuraminidase inhibitors (NAI), including oseltamivir (Dobson et al., 2015), zanamivir (Heneghan et al., 2014), and peramivir (2015), are effective against both influenza A and influenza B viruses.

## Discussion

We reviewed a number of newly developed agents systematically, with the purpose to weigh their relative advantages and limitations for utilization in the elderly population. According to the key advantages, we classified the above antibiotics into “not recommended, moderate recommended and recommend”. For example, telavancin’s better bioavailability, food-independent efficacy, and simple dosing regimens that support once daily administration, make it a potential therapy for the elderly with LRTIs.

As for nemonoxacin, all the above trials of this medicine enrolled patients over the age of 65, while this age group had not been separated into a subgroup to test the safety and efficacy of certain antibiotics alone. But considering that the elderly accounts for the majority of the participants, we still recommended nemonoxacin as a first-line medication for LRTIs in elderly according to the key criteria we have formulated above.

By contrast, zabofloxaxin has little potential for the treatment of LRTIs in elderly. Although it can be a potential therapy for COPD patients with moderate-severity exacerbations, zabofloxacin is ineffective against common non-community acquired pathogens such as aeruginosa and *A. baumannii*. For elderly patients in long-term care centers or over-hospitalized patients with underlying diseases such as cystic fibrosis, it should be noted that zabofloxacin may not be applicable (Han et al., 2013). In addition, the safety and efficacy of intravenous formulation of zabofloxacin are still unclear. Moreover, zabofloxacin has not been approved by the Food and Drug Administration (FDA) for CAP treatment.

As for telavancin, it is a novel semi-synthetic lipoglycopeptides that is active against multidrug resistant (MDR) staphylococci, enterococci, and streptococci. Telavancin’s better bioavailability, food-independent efficacy, and simple dosing regimens that support once daily administration, make it a potential therapy for old people with LRTIs.

Omadacycline was a novel once-daily aminomethylcycline antibiotic, and became the second tetracycline antibiotic approved by the FDA in 2018. Despite all the obvious advantages of omadacycline, enough attention should be given to the drawbacks for cardiac electrophysiology, namely, changes in heart rate (HR) and QT interval (Duraes and Sousa, 2019).

As a member of macrolides, solithromycin has little potential for the treatment of LRTIs in elderly. Based on the fact that solithromycin is an inhibitor of CYP3A4, the same caution should be used when co-administering solithromycin with agents that have demonstrated interaction with the precedent macrolides. Solithromycin appears to affect plasma concentrations of digoxin and warfarin, probably due to its interaction with P-glycoprotein and CYP3A4, leading to bradydysrhythmias and increased bleeding risk in elderly (Still et al., 2011). Sleep disorders and related medications, such as benzodiazepines, are commonly used in elderly, and these medications are mainly metabolized by CYP3A4 and induce side effects or attenuate therapeutic effects (Kasper and Resinger, 2001). Therefore, the combination of the two categories of agents should be avoided, and if it’s unavoidable, the dose of benzodiazepines should be reduced.

In 2010, ceftaroline was approved by the FDA and European Medicines Agency (EMA) for the treatment of CAP. Ceftaroline is only recommended in the intravenous formation in a hospitalized setting for elderly CAP patients with creatinine clearance of ≥ > 30 mL/min, no QT prolongation history, and PORT risk classes III–IV. First of all, the lack of an oral formulation for ceftaroline is a limiting property for its use in the hospital setting. Secondly, ceftaroline has weak antibacterial activity against *E*.*faecium*, *VRE*, *ESBL-E*, and *P. aeruginosa* (Kiang et al., 2015), which are common pathogens found in HAP patients with comorbidities or long-term nursing homes and are frequently treated with antibiotics. In addition, although the AEs are mostly mild, in FOCUS 1, 1.4% of ceftaroline patients and 1.0% of ceftriaxone patients developed QTcB prolongation, both of which were >500 ms, with the elongation of ≥ > 60 ms compared with the baseline.

For another Cephalosporins, ceftobiprole is suitable for patients with suspected pneumonia caused by *MRSA*, *Enterobacteriaceae* or *P. aeruginosa*, especially for patients who live in nursing homes for a long time, but more data in elderly population are required for further recommendation. Ceftobiprole, q8h, IV limits the daily activities of the elderly. For old people with malnutrition and impaired cognitive function, the risk of sarcopenia, delirium, pressure ulcer, and sputum may be increased. A survey of about one-third of clinical trial centers found that a large portion of the data in these centers were unreliable or unverifiable (Abbas et al., 2017; Jean et al., 2017), thus the FDA has requested more information and recommended additional clinical studies before ceftobiprole is approved for cSSSI and pneumonia.

We have summarized the key advantages of lefamulin in treatment of LRTIs above. In our opinion, lefamulin should be recommended as a promising agent for LRTIs in elderly, and attention must be paid to its interaction with other medicines, such as azole antifungals (Paukner et al., 2019) and midazolam (File et al., 2019) at the same time. Beyond that, it takes 12 h to the intravenous use of lefamulin, which more or less limits the activity of elderly patients and increases the possibility of convulsions.

Until now, there is insufficient evidence to support tedizolid as an ideal antibiotic therapy for LRTIs in elderly at present. Tedizolid is still not a FDA/EMA-approved antibiotic for the treatment of LRTIs, but it does bring hope to patients suffering liver and kidney organ failure, especially for LRTIs associated with linezolid-resistant Gram-positive pathogens. Although the phase 3 trial (NCT02019420) of tedizolid for HAP has not yet yielded results, tedizolid brings hope to old patients suffering renal or hepatic failure, especially with linezolid-resistant Gram-positive pathogens pneumonia (Flanagan et al., 2018).

## Conclusions

Despite noteworthy decreases in the number of deaths due to LRTIs, there remains an urgent need to make efforts to reduce the burden of disease in the elderly, especially for those with physical decline, mechanical ventilation, immunosuppression, frailty, dementia, and comorbidities. Although there are no pharmacotherapy and guidelines specifically for old patients with LRTIs, pharmacists and clinicians will need to weigh their various advantages and limitations based on the typical challenges that are faced by the elderly before choosing the optimal pharmacotherapy.

## Author Contributions

YL and BD conceived and designed the project. YL, YZ, WZ has contributed significantly to the submitted work and wrote the first draft. YL, XL, and FH revised the manuscript. All authors read and approved the final manuscript.

## Conflict of Interest

The authors declare that the research was conducted in the absence of any commercial or financial relationships that could be construed as a potential conflict of interest.
